# Influence of the Type of Bone Cement Used in Two-Stage Exchange Arthroplasty for Chronic Periarticular Joint Infection on the Spacer Replacement and Reinfection Rate

**DOI:** 10.3390/jcm12020600

**Published:** 2023-01-11

**Authors:** Meng-Wei Chang, Cheng-Ta Wu, Shih-Hsiang Yen, Timothy L. Tan, Po-Chun Lin, Feng-Chih Kuo

**Affiliations:** 1 Department of Orthopaedic Surgery, Linkou Chang Gung Memorial Hospital, Taoyuan 333, Taiwan; 2 Department of Orthopaedic Surgery, Kaohsiung Chang Gung Memorial Hospital, Kaohsiung 833, Taiwan; 3 Department of Orthopaedic Surgery, University of California, San Francisco, CA 94143, USA; 4 College of Medicine, Chang Gung University, Kaohsiung 833, Taiwan; 5 Center for General Education, Cheng Shiu University, Kaohsiung 833, Taiwan

**Keywords:** periprosthetic joint infection, commercial antibiotic-loaded bone cement, antibiotic-loaded bone cement spacer, spacer exchange

## Abstract

Background: Antibiotic-loaded bone cement (ALBC) spacers are used in the first stage when treating periprosthetic joint infection (PJI). This study aimed to investigate whether a spacer made from commercial ALBC or plain bone cement with additional antibiotics could affect the spacer exchange rate before reimplantation. Methods: Patients undergoing two-stage exchange arthroplasty due to chronic PJI from January 2014 to August 2021 were retrospectively reviewed. The exclusion criteria included arthroplasty in the setting of septic arthritis, megaprosthesis, atypical pathogen infection, spacer placement unrelated to PJI, and spacer exchange due to mechanical complications. The patient demographics, brand of cement, and microbiology were recorded manually. The primary outcome was the incidence of spacer exchange due to persistent infection and the secondary outcome was the incidence of reinfection after reimplantation. A multivariate logistic regression analysis and Chi-square test were conducted to identify the effect of cement type on the spacer exchange. Results: A total of 334 patients underwent two-stage exchange arthroplasty for PJI. The spacer exchange rates in the commercial and non-commercial ALBC groups were 6.4% and 25.1%, respectively (*p* = 0.004). After controlling for confounding factors, there were significant differences between the commercial group and non-commercial groups in the spacer exchange rate (adjusted OR = 0.25; 95% CI = 0.72–0.87, *p* = 0.029). The use of commercial ALBC was not associated with a lower reinfection rate after reimplantation (*p* = 0.160). Conclusions: In a two-stage exchange arthroplasty scenario, the spacer comprised of commercial ALBC resulted in a lower spacer exchange rate than the plain bone cement, both of which had additional antibiotics. However, the use of commercial ALBC was not associated with a lower incidence of reinfection following reimplantation.

## 1. Introduction

Two-stage exchange arthroplasty remains the standard treatment for chronic periprosthetic joint infection (PJI) [1]. The first stage aims at controlling infection through the removal of the infected prosthesis and the insertion of an antibiotic-loaded cement spacer, followed by a 6 to 8 weeks systemic course of antibiotics. Reimplantation is then performed when the infection is believed to be controlled [2,3]. However, up to 17% of patients receive an additional spacer exchange due to persistent infection [4]. Patients with a spacer exchange have also consistently demonstrated a higher reinfection rate following reimplantation [4].

Acrylic bone cements are extensive used in the area of orthopedic surgery, including vertebroplasty, primary or revision joint arthroplasty, bone defect filling, and as carriers of antibiotics. Buchholz and Engelbrecht first introduced antibiotics-loaded bone cement, composed of polymethyl methacrylate (PMMA) and its monomer, in the 1970s [5]. Even though the mechanical strength will be affected by an improper mix ratio or environment and additional biodegradable materials, such as different types of antibiotics, antibiotic-loaded bone cement still remains important in clinical usage [6,7,8,9].

An antibiotic-loaded bone cement (ALBC) spacer, either static or mobile, plays an important role in the first stage of a two-stage exchange by providing both structural support and elution of antibiotics [10]. The amount and the duration of the release of the antibiotic from ALBC are influenced by a variety of factors, including the concentration and number of antibiotics [11] and the cement type [11,12]. Generally, surgeons manually mix additional high-dose antibiotics into commercial ALBC or plain bone cement in the form of a spacer or beads. While in vitro studies have demonstrated that commercial ALBC eluted a higher concentration of local antibiotics than when the antibiotics were hand-mixed in plain bone cement [13,14], there is little clinical evidence regarding which is superior. Furthermore, one disadvantage of commercial ALBC is that it is associated with increased costs. Therefore, it is important to clarify where the use of commercial ALBC provides any clinical benefit compared with bone cement.

The purpose of this study was to evaluate whether a spacer made by commercial ALBC can decrease the spacer exchange after the first stage of a two-stage exchange compared with those made by plain bone cement with the addition of hand-mixed antibiotics.

## 2. Materials and Methods

**Figure 1:**
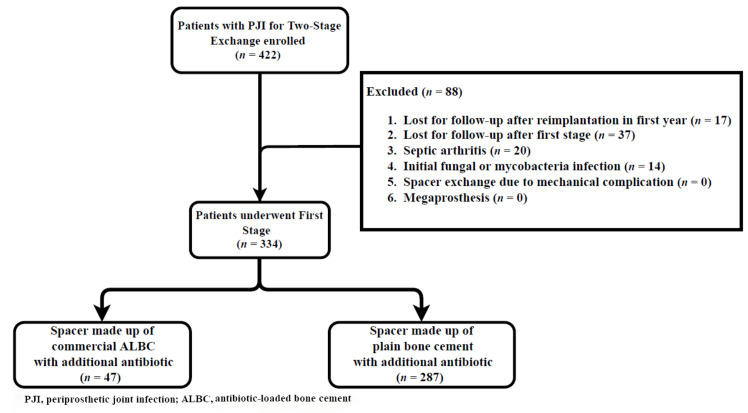
Diagram of patient enrollment and exclusion.

Following institutional review board approval, a retrospective single institutional study was performed to identify 422 chronic periprosthetic joint infections (PJIs) in patients who underwent two-stage exchange arthroplasty from January 2014 to August 2021. The diagnosis of PJI was based on the definition from the 2018 International Consensus Meeting [15]. We excluded patients who were lost to follow-up after reimplantation in the first year, those who were lost to follow-up after the first stage, and those with native septic arthritis. Patients with an atypical infection, including fungus and mycobacteria infection, spacer exchange due to a mechanical complication, and those with a megaprosthesis were also excluded. After 88 patients were excluded because of the aforementioned criteria, a total of 334 patients (157 knees and 177 hips) with chronic PJI fulfilled the inclusion and exclusion criteria, including 47 patients with commercial ALBC and 287 patients with non-commercial bone cement at the first stage of a two-stage exchange (Figure 1).

### 2.1. Surgical Technique

In the first stage of a two-stage exchange, the removal of the prosthesis was performed in addition to radical debridement, and betadine lavage was diluted. An antibiotic-loaded cement spacer, either static or mobile, was constructed and placed. Multiple tissue cultures (≥3) were routinely obtained during the operation. We used three types of commercial ALBC, including Antibiotic Simplex™ P cement with tobramycin, with a medium viscosity and 41 g powder including 1 g tobramycin (Stryker, Kalamazoo, MI, USA); Palacos^®^ MV + G cement with gentamycin, with a medium viscosity and 45.2 g powder including 0.6 g gentamycin (Heraeus Medical LLC, Yardley, PA, USA); and Vancogenx^®^ with vancomycin and gentamycin, with a medium viscosity and 40 g powder including 1 g gentamycin and 1 g vancomycin (Tecres, Verona, Italy). The plain bone cement that was utilized was Simplex™ P bone cement (Stryker, Kalamazoo, MI, USA), with a medium viscosity and 40 g powder. The choice of a commercial ALBC or plain bone cement was based on the patient’s financial status, including an affordable medical fee for patients, insurance coverage, and family support, as well as the surgeon’s preference. Even though commercial ALBC was composed of cement powder and antibiotics, such as gentamycin, vancomycin, or tobramycin, they still could not provide broad spectrum coverage of the microorganism. Therefore, per 40 g of bone cement, either commercial ALBC or plain bone cement, we normally added 2–4 g of vancomycin and 2–4 g of ceftazidime (standard antibiotic regimen) providing broad spectrum coverage and accounting for 74% (248/334) [16]. The other antibiotics we selected were targeted toward the culture results, including gentamycin, daptomycin, piperacillin/tazobactam, etc.

After spacer implantation, the patients received at least 6 weeks of systemic antibiotics treatment, which was sufficient to control PJI before reimplantation [17,18]. The regimen of systemic antibiotics was based on the susceptibility profile in previous culture results and the recommendations of infectious diseases experts. No single marker could give us a hint of whether to undergo reimplantation. Therefore, patients in which persisted infection was suspected, based on the 2018 International Consensus Meeting for PJI, including tissue culture, synovial fluid PMN%, elevated C-reactive protein (CRP) and erythrocyte sedimentation rate (ESR), poor wound integrity, and purulence drainage, would receive a spacer exchange and continued systemic antibiotic treatment [10,16,19]. Reimplantation was performed when the above signs were not observed. 

Data were collected through electronic medical records, including patient demographic factors (gender, age, and BMI), joint involvement (hip or knee), laterality, type of bone cement (either commercial or non-commercial), type of spacer (static or mobile), local antibiotic dose in the bone cement, American Society of Anesthesiologists (ASA) grade, serum erythrocyte sedimentation rate (ESR) and C-reactive protein (CRP), and microorganism report at the spacer exchange.

### 2.2. Outcome Assessment

The primary outcome was the incidence of spacer exchange due to persistent infection before prosthetic joint reimplantation and the secondary outcome assessed the reinfection rate after reimplantation [20].

### 2.3. Statistical Analysis

Chi-squared and Kruskal–Wallis rank tests were performed for categorical variables and continuous variables between the commercial and non-commercial groups. Because of the different types and doses of additional antibiotics, the formula of standardization, $x^{\prime }=\frac{x-\bar{x}}{\sigma }$, was used to standardize every different kind of antibiotics and then summed them up. After being divided by the packs of cement, it was used in a logistic regression model. To further investigate whether the utilization of commercial ALBC is associated with the rate of spacer exchange, the covariates with a *p*-value less than 0.2 in the univariate analysis were added to a multivariate logistic regression model. Post hoc power analysis using the difference between two dependent means was performed on the spacer exchange rate during two-stage exchange arthroplasty to determine the likelihood of a type 2 error (missing a significant difference between the commercial group and the non-commercial group when one in fact exists). Based on the current information (beta = 0.897), the sample size was adequately powered at 89.7% to detect the difference between the treatment group. The results were presented as odds ratios (ORs) with 95% confidence intervals (CIs). Medcalc^®^ Version 19.5.3 (MedCalc Software Ltd., Ostend, Belgium), and SPSS Version 26 (IBM, SPSS Inc., Chicago, IL, USA) were utilized for the statistical analysis.

## 3. Results

There was no significant difference between commercial and non-commercial groups regarding age, gender, BMI, laterality, spacer type, ASA, culture result (polymicrobial and resistant organism), serum CPR and ESR, standard antibiotic regimen, and mean standardized hand-mixed antibiotics per cement (Table 1). However, the proportion of knee to hip in the commercial group was significantly higher than in the non-commercial group (*p* = 0.029).

**Figure 2:**
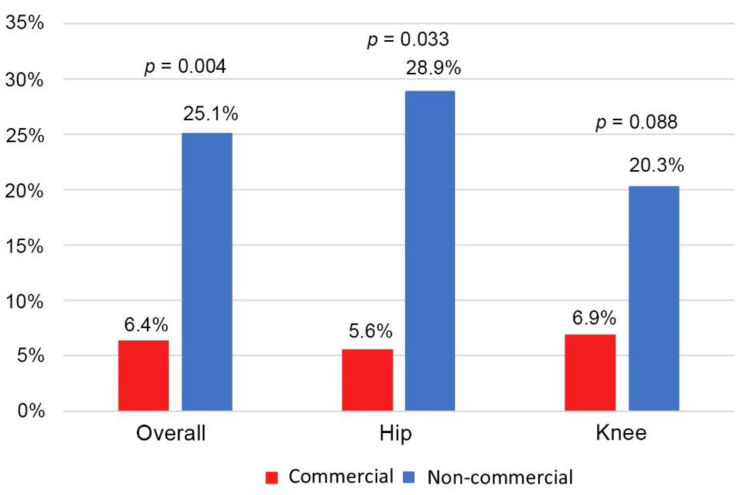
The spacer exchange rate between the hips and knees.

The overall spacer exchange rate was 22.4% (75/334). In Figure 2, the overall spacer exchange rate in the commercial bone cement group was significantly lower than the non-commercial bone cement group (*p* = 0.004). Among the subgroup analysis of hip PJI, the spacer exchange rate in the commercial bone cement group was significantly lower than non-commercial bone cement groups (*p* = 0.033). In the knees, the spacer exchange rate was decreased in the commercial bone cement group, but was not statistically significant (*p* = 0.088).

In the univariate analysis we used the commercial ALBC, and age, joint, spacer type, polymicrobial, serum CRP, and ESR were significantly correlated to the spacer exchange (Table 2). After controlling for the potential confounders, commercial ALBC was significantly associated with a lower spacer exchange (adjusted OR = 0.25; 95% CI = 0.72–0.87, *p* = 0.029). The other covariates remained statistically significant, including age (adjusted OR = 0.95; 95% CI = 0.93–0.98, *p* < 0.001), mobile spacer (adjusted OR = 0.42; 95% CI = 0.23–0.74, *p* = 0.003), polymicrobial (adjusted OR = 3.60; 95% CI = 1.38–9.37, *p* = 0.009), and serum ESR (adjusted OR = 1.01; 95% CI = 1.00–1.02, *p* = 0.031).

**Figure 3:**
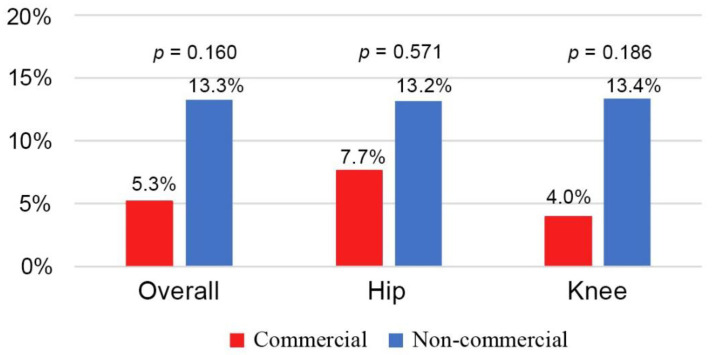
The reinfection rate after reimplantation between the hips and knees.

The total reimplantation rate was 84% (279/334). The overall reinfection rate after reimplantation was 12.2% (34/279). The reinfection rate was 5.3% in the commercial cement group and 13.3% in the non-commercial cement group (*p* = 0.160, Figure 3). There was no significant difference in the reinfection rate between the commercial and non-commercial cement groups when stratified by hips and knees. For revision hip, the reinfection rate was 7.7% in the commercial cement group and 13.2% in the non-commercial cement groups (*p* = 0.571). For revision knee, the reinfection rate was 4.0% and 13.4% in the commercial and non-commercial cement groups (*p* = 0.186), respectively. There was no significant difference between the two groups regarding the microorganism (Table 3).

## 4. Discussion

Our study demonstrates that the use of commercial ALBC did result in a lower spacer exchange rate than the plain bone cement, both with manually adding additional antibiotics, during two-stage exchange arthroplasty for treating periprosthetic joint infection. The result was consistent after a multivariate logistic regression. The subgroup analysis of the hip PJI also shows that the spacer exchange rate was lower in the commercial ALBC group. In this study, the total spacer exchange rate was 22.4% compared with 11% to 30% in the literature [4,21,22]. However, George et al. [21] only recorded the patients that were reimplanted, which could underestimate the spacer exchange rate by not including patients with retained spacers. Furthermore, Corona et al. [22] and Tan et al. [4] reported that 11% (*n* = 18/162) and 30% (*n* = 27/90) of patients with the spacer exchange did not go through reimplantation.

The ALBC spacer can deliver a high concentration of local antibiotics at a therapeutic level [23]. Many factors affect the elution of antibiotics from the ALBC spacer [24]. Anagnostakos et al. proposed that antibiotic release is increased with porosity, which is affected by the cement type, antibiotic type, and combinations [25]. The plain bone cement we used in this study was Simplex P cement. Meeker et al. demonstrated that Simplex P cement, mixed with vancomycin, had the lowest antibiotic level compared with the other three different brands of bone cement, including Palacos LV, BIOMET Cobalt HV, and Zimmer Biomet Bone Cement R [26]. The commercial ALBCs we used to be three brands: Antibiotic Simplex™ P cement with tobramycin [Stryker, Kalamazoo, MI], Palacos^®^ MV + G cement (Heraeus Medical LLC, Yardley, PA, USA), and Vancogenx^®^ with vancomycin and gentamycin (Tecres, Verona, Italy). Lee et al. also proposed that Palacos had the most significant release amount of vancomycin compared with Simplex P, Osteobond, and Depuy CMW in an in vitro study [27]. The release of antibiotics from ALBC also depends on the surface roughness and bulk porosity. The initial release rate was related to the surface roughness, and the total release amount of antibiotics was influenced by the porosity [28]. Frew et al. demonstrated that manual additional antibiotics in the bone cement could increase the porosity and surface area so as to achieve a better total elution amount of antibiotics than the commercial ALBC [24]. In our study, both commercial and non-commercial groups contained manually added antibiotics, including vancomycin, gentamycin, tobramycin, and ceftazidime [16], into commercial ALBC or plain bone cement.

The amount of implanted cement was not considered in our study because it was difficult to weigh every spacer in the clinical setting. For example, the ALBC spacer would be shaped to fit the size of the joint. Therefore, we used the mean standardized hand-mixed antibiotics per pack of cement as the concentration of spacer to analyze the effect of the additional antibiotics in our study. Undoubtedly, the amount of antibiotics influenced the antibiotic elution. However, Duey et al. reported that the release of antibiotics was correlated with the specimen’s surface area compared with the specimen’s volume [29]. Furthermore, Marsi et al. demonstrated that the surface area of the cement had a remarkable effect on the elution of antibiotic-loaded bone cement [30]. Schurman et al. demonstrated that 81% of antibiotics were eluted from a superficial layer of cement [31]. Even if the total cement volume was decreased, elution antibiotics had no significant change when the surface area remained the same.

According to our study, the mobile or dynamic spacer resulted in a lower spacer exchange rate than the static spacer. Belt et al. reported that the surface roughness and bulk porosity would affect the release of antibiotics from ALBC [32]. Evan et al. demonstrated that the articular spacer had a high coefficient of friction that led to wear debris [32]. Therefore, both the surface roughness and the release of antibiotics would increase because of the high coefficient of friction. George et al. also reported that articulating spacers showed a better infection eradication rate than the static spacers [33]. Moreover, the mobile spacer could provide a positive effect for maintaining joint motion, preserving the extensor mechanism, and improving post-operative function. Better post-operative ROM after second-stage procedure with articulating spacers achieving 107.8 degrees over 93.7 degrees in static spacers was noted by Emerson et al. [34]. Hofmann et al. also demonstrated improved post-operative motion and pain by using an articulating spacer.

Some studies reported polymicrobial PJI accounting for 6 to 37%. Compared with the monomicrobial PJI, a lower cure rate was also noted in the polymicrobial PJI [35,36,37,38]. Our study showed that the incidence of polymicrobial was about 7.5% in our study, and was significantly associated with a high spacer exchange rate. Polymicrobial was often associated with chronic infection and produced lots of synergy advantages to form the biofilm, including passive resistance, metabolic cooperation, and an enlarged gene pool [39]. Synergy could also stimulate resistance and suppress the immune system via commensal bacteria [40]. Because of the aforementioned effect, prolong infectious status, elevated ESR, and even the failure of the eradication of infection was revealed in a clinical setting. In addition, Tan et al. demonstrated that polymicrobial PJI was associated with a high amputation, arthrodesis, and PJI-related mortality [38].

The reinfection rate in our study was 12.2% (34/279), which was in accordance with other reports in the literature. Wasielewski et al. reported 10% of patients had a reinfected knee after two-stage exchange arthroplasty [41]. Kubista et al. demonstrated that the incidence of reinfection was 15.8% after reimplantation [42]. Several risk factors have been mentioned in previous studies. Petis et al. revealed that a previous revision operation was a risk factor for reinfection [2]. Hartman et al. reported elevated CRP levels at the time of diagnosis and methicillin-sensitive *Staphylococcus aureus* infection were associated with the reinfection rate [43]. Logroscino et al. revealed obesity (BMI > 25) and multiple previous procedures as risks [44]. Our study showed that the use of commercial ALBC did not result in a lower reinfection rate than for plain bone cement, for both with manually added antibiotics. Before reimplantation, both the plain bone cement and commercial ALBC could control infection well. Therefore, the type of cement was not associated with the reinfection rate.

Antibiotic-loaded bone cement could reach a higher concentration with less systemic host toxicity and a more constant concentration among the surrounding tissue than parenteral antibiotic treatment [45,46]. Salvati et al. reported that a total of 56 cases of gentamycin-loaded bone cement (38 cases) and beads (18 cases) that demonstrated low serum and urine gentamycin levels that showed no systemic toxic effects [47]. In addition, Springer et al. found no observed systemic toxicity in 36 cases of PJI using a high dose of vancomycin and gentamycin in ALBC spacers [48].

The strengths of this study include that we documented the reasons for spacer exchange thoroughly and excluded cases of spacer exchange due to mechanical complications. Furthermore, atypical pathogen infections, such as fungus and mycobacterium infection, were also excluded, which allowed us to isolate the antibiotic efficacy against bacterial infection. The main limitation of this study lies in the retrospective nature of the study, including brands of commercial ALBC, the spacer type, and the degree of tissue debridement. Moreover, the selection of additional antibiotics could not be unified. The empirical choice was mainly based on previous research, including vancomycin and ceftazidime [16]. Secondly, the decision to perform a spacer exchange for persistent infection was often based on the surgeon’s discretion, as there are no clear metrics to determine the timing for reimplantation. Third, the commercial cement we used in this study consisted of three different brands, namely Stryker, Heraeus, and Tecres, which added heterogeneity to the study. Fourth, the mechanical strength was not tested in our study between the two groups. However, there was no spacer exchange due to spacer fracture. The last limitation of this study was that the viscosity of cement used in our study was medium. We could not define the effect of different viscosity (low, medium, and high) on the release of antibiotics, which had a role in the release of antibiotics from ALBC [25]. Furthermore, the lack of difference in the results of the study could be due to a lack of power, as there was not a large sample size of patients with commercially available ALBC.

## 5. Conclusions

In our study, the usage of commercial ALBC with the manual additional antibiotic did reduce the spacer exchange rate compared with the non-commercial cement following the first stage of a two-stage exchange arthroplasty in hip and knee PJI. Further studies, either prospective or randomized control studies, are still required to confirm our findings and to clarify the relationship between commercial ALBC and mechanical strength in a clinical setting.

## Figures and Tables

**Table 1:** Patients’ demographics between the two groups.

Variable	Commercial Cement Group (*n* = 47)	Non-Commercial Cement Group (*n* = 287)	*p* Value
Median age, year (IQR)	69 (16)	66 (19)	0.053
Sex, *n* (%)			0.454
Male	20 (43%)	139 (48%)	
Female	27 (57%)	148 (52%)	
Median BMI, kg/m^2^ (IQR)	25.6 (5.96)	25.4 (6.02)	0.748
Joint, *n* (%)			0.029 *
Hip	18 (38%)	159 (55%)	
Knee	29 (62%)	128 (45%)	
Laterality, *n* (%)			0.235
Left	21 (45%)	141 (49%)	
Right	26 (55%)	146 (51%)	
Spacer type, *n* (%)			0.965
Mobile	28 (60%)	170 (59%)	
Static	19 (40%)	117 (41%)	
ASA, *n* (%)			0.622
1	1 (2%)	4 (2%)	
2	15 (32%)	112 (38%)	
3	30 (64%)	169 (59%)	
4	1 (2%)	2 (1%)	
Polymicrobial, *n* (%)			0.364
No	45 (96%)	264 (92%)	
Yes	2 (4%)	23 (8%)	
Resistant organism, *n* (%)			0.844
No	43 (92%)	260 (91%)	
Yes	4 (8%)	27 (9%)	
Median Serum CRP, mg/L (IQR)	33.6 (55)	38.2 (97)	0.155
Median Serum ESR, mm/h (IQR)	68 (65)	65 (58)	0.596
Standard antibiotic regimen ^†^, *n* (%)	29 (62%)	200 (70%)	0.275
Mean standardized hand-mixed antibiotics per cement (IQR)	2.2 (1.1)	1.9 (1.5)	0.279

* Chi-squared test, and Kruskal–Wallis Rank Test with α = 0.05 being significant. BMI, body mass index; IQR, interquartile range; ASA, American Society of Anesthesiology; CRP, C-reactive protein; ESR, erythrocyte sedimentation rate. ^†^ Vancomycin and ceftazidime as the standard antibiotic regimen.

**Table 2:** Univariable and multivariable analysis of the spacer exchange for periprosthetic joint infection.

Covariables	Univariable Analysis			Multivariable Analysis		
	OR	95% CI	*p*-Value	OR	95% CI	*p*-Value
Cement group						
Non-commercial	Ref			Ref.		
Commercial	0.19	0.05–0.83	0.027 *	0.25	0.72–0.87	0.029 *
Age	0.96	0.94–0.98	<0.001	0.95	0.93–0.98	<0.001
Gender	0.88	0.55–1.47	0.634	-	-	-
BMI	0.98	0.93–1.03	0.438			
Joint						
Hip	Ref.			-	-	-
Knee	0.58	0.35–0.99	0.045 *	-	-	-
Laterality	0.99	0.59–1.65	0.971	-	-	-
BMI	0.98	0.93–1.03	0.440	-	-	-
Spacer type						
Static	Ref.			Ref.		
Mobile	0.50	0.30–0.84	0.008 *	0.42	0.23–0.74	0.003 *
Polymicrobial	2.46	1.05–5.72	0.037 *	3.60	1.38–9.37	0.009 *
ASA						
1	Ref.					
2	0.20	0.03–1.24	0.083	-	-	-
3	0.18	0.03–1.10	0.064	-	-	-
4	1.33	0.07–26.6	0.851	-	-	-
Resistant organism	1.20	0.52–2.81	0.671	-	-	-
Serum CRP	0.50	0.30–0.84	0.008 *	1.00	0.99–1.01	0.064
Serum ESR	1.01	1.00–1.02	0.035 *	1.01	1.00–1.02	0.031 *
Standard antibiotic regimen	1.48	0.87–2.52	0.152	-	-	-
Mean standardized hand-mixed antibiotics per cement	0.99	0.90–1.11	0.965	-	-	-

* Logistic regression with α = 0.05 as significant. OR, odds ratio; CI, confidence interval; BMI, body mass index; ASA, American Society of Anesthesiology; CRP, C-reactive protein; ESR, erythrocyte sedimentation rate.

**Table 3:** Microorganism profile at the spacer exchange between the groups.

Microorganism	Commercial Cement Group	Non-Commercial Cement Group	*p*-Value
*Staphylococcus aureus*	0	8	0.606
Coagulase-negative staphylococci	1	3	0.456
Streptococcus species	0	2	1.000
Methicillin-resistant *Staphylococcus aureus*	0	7	1.000
Gram negative bacteria	1	9	1.000
Polymicrobial	0	11	0.374

## Data Availability

The data presented in this study are available on request from the corresponding author.
